# Altered Cogs of the Clock: Insights into the Embryonic Etiology of Spondylocostal Dysostosis

**DOI:** 10.3390/jdb9010005

**Published:** 2021-01-29

**Authors:** Ana Nóbrega, Ana C. Maia-Fernandes, Raquel P. Andrade

**Affiliations:** 1 CBMR, Centre for Biomedical Research, Universidade do Algarve, 8005-139 Faro, Portugal; anacbsn@gmail.com (A.N.); ac.maia.fernandes@gmail.com (A.C.M.-F.); 2 Faculdade de Medicina e Ciências Biomédicas (FMCB), Universidade do Algarve, Campus de Gambelas, 8005-139 Faro, Portugal; 3 ABC-RI, Algarve Biomedical Center Research Institute, 8005-139 Faro, Portugal; 4 Champalimaud Research Program, Champalimaud Center for the Unknown, 1400-038 Lisbon, Portugal

**Keywords:** spondylocostal dysostosis, somitogenesis clock, somite formation, HES7, LFNG, DLL3, MESP2, TBX6, RIPPLY2

## Abstract

Spondylocostal dysostosis (SCDO) is a rare heritable congenital condition, characterized by multiple severe malformations of the vertebrae and ribs. Great advances were made in the last decades at the clinical level, by identifying the genetic mutations underlying the different forms of the disease. These were matched by extraordinary findings in the Developmental Biology field, which elucidated the cellular and molecular mechanisms involved in embryo body segmentation into the precursors of the axial skeleton. Of particular relevance was the discovery of the somitogenesis molecular clock that controls the progression of somite boundary formation over time. An overview of these concepts is presented, including the evidence obtained from animal models on the embryonic origins of the mutant-dependent disease. Evidence of an environmental contribution to the severity of the disease is discussed. Finally, a brief reference is made to emerging in vitro models of human somitogenesis which are being employed to model the molecular and cellular events occurring in SCDO. These represent great promise for understanding this and other human diseases and for the development of more efficient therapeutic approaches.

## 1. Introduction

Biomedical research employs multiple scientific inquiry approaches, such as basic, clinical, and translational research and great advances have been made in any one of these fronts. The ultimate aim is to have such a clear understanding of the molecular and cellular causes of the phenotypical and clinical manifestations of Human diseases, so that a successful therapeutic solution or preventive strategy may be developed. Developmental Biology has greatly contributed to understanding the pathophysiology of multiple congenital diseases. Congenital malformations, defined as structural or functional abnormalities that are present since birth, are globally responsible for 11% of newborn deaths [1]. These are largely due to genetic causes; however, phenotypic variability also depends on epigenetic components and environmental factors to which the individual is exposed during embryo development [2]. Within congenital malformations, spinal dysplasias occur in about 1 in 5000 births [3] and spondylocostal dysostosis (SCDO), considered a rare genetic disease, has an estimated incidence of 1 in every 40,000 births [4].

This work aims to reflect the current evidence regarding the embryonic etiology of SCDO. Data obtained from clinical, genetic, and developmental biology studies are steadily closing the gaps on the comprehension of this rare, albeit severe congenital disease. Initially, a summary of the phenotypic and genetic characteristics of SCDO is presented. This is followed by the current knowledge on the embryonic processes leading to the formation of the axial skeleton, with a special focus on the molecular mechanisms underlying somite formation and the somitogenesis molecular clock. The contribution of animal models for evidence of a causal relationship between mutations in the genes involved in somitogenesis and the development of SCDO are presented. Finally, the newly emerging in vitro models to study the human somitogenesis clock and SCDO onset are discussed.

## 2. Spondylocostal Dysostosis

The term Spondylocostal dysostosis (SCDO) includes an etiologically heterogeneous group of heritable diseases characterized by congenital malformations of the axial skeleton at the level of the vertebrae and ribs, with varying degrees of severity. In 1938, Saul Jarcho and Paul M. Levin made the first description of what is currently classified as Type 2 SCDO [5]. For many years thereafter, the Jarcho-Levin Syndrome classification covered any individual with congenital defects in vertebrae segmentation and rib abnormalities, even if only remotely related to the syndrome originally described [6]. 

Phenotypically, SCDO patients are characterized by a compressed trunk that is disproportionately short relative to the individual’s total height, a short and rigid neck, and protrusion of the abdomen, often accompanied by moderate and non-progressive scoliosis [6]. Typically, segmentation defects occur in 10 contiguous vertebrae. Respiratory complications are among the most common causes of morbidity and mortality in affected individuals; the restrictions imposed by the deformation of the rib cage affect lung development and signs and symptoms such as tachypnea, fatigue or lung infections are often present as a result of pulmonary hypoplasia [7] and abnormal chest compliance [8]. SCDO usually occurs in isolation, with manifestation of abnormalities restricted to the spine and ribs [6]. A somewhat similar, albeit a clinically and radiologically independent disease, is spondylothoracic dysostosis (STD), first described by Lavy, Palmer, and Merritt in 1966 [4,9]. STD’s main distinguishing feature is a shorter but symmetrical chest, with fused thoracic vertebrae, and overall-normal ribs that are fused next to their vertebral origin acquiring a fan-like or crab-like configuration. STD is often associated with early death from respiratory dysfunction or infection [4,8].

**Figure 1:**
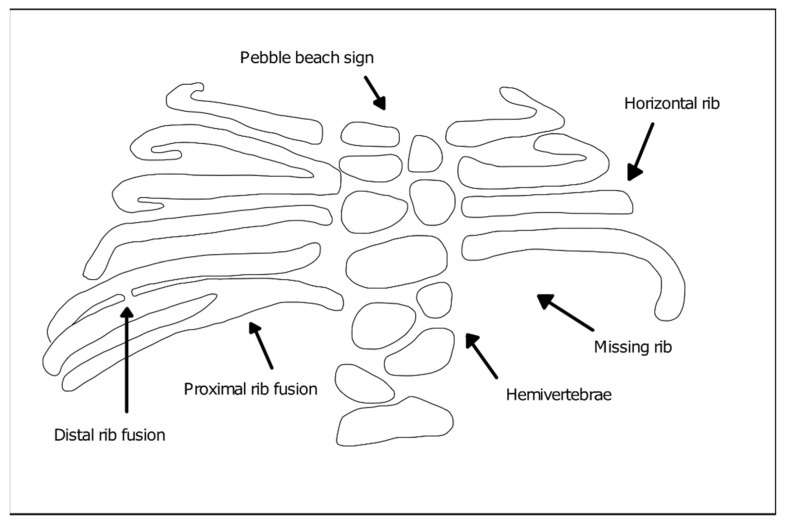
Vertebral and rib malformations identified in different types of spondylocostal dysostosis (SCDO).

The diagnosis of SCDO is based on the clinic and the radiological images. Radiological findings are diversified and include the involvement of several segments of the spine, especially in the thoracic region, which present butterfly-like vertebrae, fused hemivertebrae or vertebrae, and also diverse alterations to rib morphology (Figure 1). These include rib enlargement, fusion, bifurcation or even agenesis, causing asymmetries with incorrect rib alignment and often with a reduction in the total number of ribs [7]. The identification of the specific disease type is confirmed by genetic tests to identify mutant alleles in one of the seven currently known genes responsible for the different types of SCDO [6].

### Genetics and Main Phenotypic Characteristics of SCDO Subtypes

SCDO is a genetic disease with an autosomal inheritance pattern that is predominantly recessive [10]. The disease is most common in cases of consanguinity of the parents and is associated with the transmission of pathological mutations in genes with a key role in the formation of the axial skeleton during embryogenesis. The SCDO-associated genes known to date and their associated SCDO subtypes are the following:

- DLL3 (Delta-like protein 3)—Type 1 SCDO (OMIM #277300). Type 1 SCDO is the most common form found in clinical practice and the majority of the affected individuals result from inbreeding unions [6]. DLL3 mutations identified in patients include insertions, frameshift, splicing, and nonsense mutations leading to premature truncation or protein function impairment [11,12]. Phenotypically, the mutation of this gene leads to moderate, non-progressive scoliosis and rarely requires surgical intervention to stabilize the spine. The affected individuals consistently show an irregular ossification pattern, with the vertebral bodies assuming a rounded or oval shape during childhood (“pebble beach sign”), evolving into irregular vertebral bodies and hemivertebrae as ossification is completed [6];
- MESP2 (Mesoderm posterior protein 2)—Type 2 SCDO (OMIM #608681). The pathogenic variant of this gene results in straight ribs with fewer fusion points and therefore, more regularly aligned when compared to other types. SCDO type 2 has been described in three families, one of them with consanguineous parents [6]. Reported MESP2 missense mutations introduce premature stop codons leading to protein truncation [13]. Other mutations thought to severely reduce protein levels due to nonsense-mediated mRNA decay, are found in cases of STD and are associated with more severe phenotypes [6,13];
- LFNG (Lunatic Fringe)—Type 3 SCDO (OMIM #609813). There are two reports of LFNG mutations associated with SCDO. The first documented individual presented a more severe shortening of the spine than that found in the other SCDO subtypes, with all vertebral bodies exhibiting severe segmentation defects. Rib anomalies were similar to those in SCDO type 1 and 2 [14,15]. An additional report was made of an individual carrying two distinct mutations in LFNG, with multiple vertebral defects along the entire spine [16,17]. In both reported cases, the identified missense mutations were found to impair Lfng enzymatic activity and/or subcellular localization [17]. Comparable vertebral malformations were described in individuals with mutations in the SLC35A3 gene encoding the Golgi UDP-GlcNAc transporter [18] (required for Lfng substrate availability), which further supports the importance of Lfng activity in axial skeleton formation;
- HES7 (Hairy enhancer of split 7)—Type 4 SCDO (OMIM #613686). HES7 belongs to the family of hairy-enhancer-of-split transcription factors and is specifically expressed in the embryonic paraxial presomitic mesoderm (PSM) [19]. Mutations in HES7 were described in infants presenting a shortened spine, with segmentation defects predominantly in the thoracic region and irregularly aligned, fused ribs [20,21]. The identified missense mutations resulted in significant reduction of HES7 transcriptional inhibitory activity and alterations to its heterodimerization potential [20,21]. In some cases, neural tube closure defects were also present, although there is no evidence of a direct association of the two conditions. Type 4 SCDO was described in three families, one of which also had inbreeding [6];
- TBX6 (T-box transcription factor 6)—Type 5 SCDO (OMIM #122600). SCDO-associated mutations in TBX6 were described in three generations of the same family, following an autosomal dominant inheritance. The affected individuals, all male, had a mixture of hemivertebrae and blocks of fused vertebral segments, moderate scoliosis affecting the middle thoracic region, with little involvement of the ribs [22,23]. Multiple other cases have also been reported and the underlying TBX6 mutations include 16p11.2 genomic deletions, as well as nonsense and frameshift mutations, some of which were found to alter TBX6 subcellular localization and/or transcriptional activity [16,24,25];
- RIPPLY2 (Protein ripply 2)—Type 6 SCDO (OMIM #616566). The first report of mutations in this gene described two brothers who had vertebral segmentation defects in cervical and thoracic regions, including hemivertebrae and butterfly vertebrae but overall normal ribs, with marked cervical kyphosis and moderate thoracic scoliosis [6,26]. One of the reported mutations introduces a premature stop codon, with consequent loss of transcriptional repressor activity; the other is a missense mutation localized at a mRNA splice site, but its functional consequences have not yet been elucidated [26]. Since then, RIPPLY2 mutations were described in several other individuals with vertebral defects, many times associated with additional congenital malformations [27,28];
- DMRT2 (Doublesex And Mab-3 Related Transcription Factor 2; OMIM *604935). An homozygous DMRT2 variant, predicted to lead to the absence of full length DMRT2 protein product due to loss of the start codon, was recently associated with a severe form of a SCDO-like phenotype [29]. The newborn presented severe costovertebral defects, with all ribs affected either in size or shape (missing, fused, bifid, and hypoplastic), particularly in the most distal part. The vertebrae were also malformed (laminae intervertebral fusions and irregular ossification), despite the absence of clear segmentation defects of the vertebral bodies.

Despite the growing knowledge of the clinical and genetic aspects associated with SCDO, significant challenges still remain in understanding the underlying pathological mechanisms. Developmental engineering is a promising area in terms of treatment strategies for diseases related to embryonic development of the spine. Its success however depends on the ever-growing knowledge of the disease’s embryonic etiology together with the embryonic developmental processes affected [30].

## 3. Formation of the Spine during Embryogenesis

The human axial skeleton is an extraordinarily structured system that starts to form early in embryo development. Soon after blastocyst implantation in the uterus, gastrulation takes place giving rise to the three embryonic germ layers: ectoderm, mesoderm, and endoderm. The PSM, located bilaterally to the axial neural tube, is then segmented into round-shaped transient structures called somites. As new somite pairs are periodically formed in the anterior region, new cells are added to the posterior PSM, allowing somitogenesis to progress simultaneously with axial extension (reviewed in [31,32]). Between the 20th day and the 5th week of gestation, a total of 42 to 44 pairs of somites are formed, with each pair of somites appearing every 5 h [33,34]. Temporal precision of somitogenesis is observed in all vertebrates, with species-specific periodicities, such as 90 min in the chicken and 2 h in the mouse embryo (reviewed in [31]). Later, somites will differentiate into vertebrae, ribs, and the skeletal muscles that provide stability and movement to the axial skeleton.

### 3.1. The Somitogenesis Molecular Clock 

**Figure 2:**
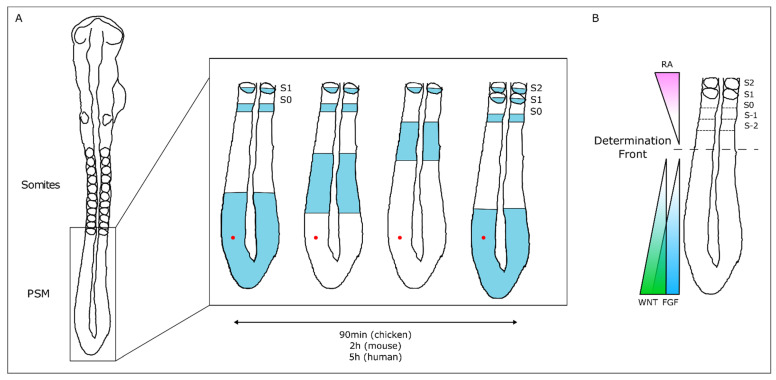
The Molecular Clock and Signaling Gradients in temporal/spatial control of vertebrate somitogenesis. (**A**) Representation of one oscillation of *hairy1* gene expression (blue) in the presomitic mesoderm (PSM) of a 48h chicken embryo. A cell in the PSM (red dot) undergoes a complete cycle of gene expression activation-repression-activation as a new somite is formed in the anterior-most PSM. (**B**) Opposing gradients of retinoic acid (RA) and WNT/FGF signaling converge at the determination front; rostrally, the PSM tissue is already committed to form somites.

The temporal and spatial precision of somitogenesis has long intrigued many scientists. One of the first theoretical models to explain how this process is regulated—the Clock and Wavefront model—proposed the existence of a molecular oscillator (Clock) that would define the tempo of somite formation and of a Wavefront of cell differentiation, that would set the position of each new somite boundary [35]. The experimental evidence of such a molecular oscillator was provided 20 years later, when Palmeirim et al. described *hairy1* gene expression oscillations in the chicken PSM with the same periodicity as somite formation, 90 min [36] (Figure 2). Since then, many genes with oscillatory expression in the PSM were found in multiple vertebrate species, suggesting that this is a conserved feature of vertebrate embryos [37]. Strikingly, the clock periodicity in each species is directly coupled to the time required to form a pair of somites. This is 90 min for the chicken embryo, 2 h in the mouse, and approximately 5 h in humans. 

The wavefront proposed in the Clock and Wavefront model [35] plays an important role in the correct positioning of each new somite boundary in the anterior-end of the PSM [38]. An anterior retinoic acid (RA) signaling gradient opposes posterior-derived Fgf and Wnt gradients, establishing the so-called determination front, where PSM cells become irreversibly committed to form a somite within the next 3–4 molecular clock oscillations [38,39,40]. Posterior-to-anterior gradients of Wnt and Fgf signaling activity maintain cells in an undifferentiated state in the posterior PSM. Overexpressing Fgf8 at the level of the determination front leads to the formation of smaller somites, while if Fgf signaling is inhibited, larger somites are formed [38]. Moreover, both Fgf and Wnt signaling are crucial for embryo elongation, thus coupling the process of somitogenesis with anterior-to-posterior elongation [41,42]. 

**Figure 3:**
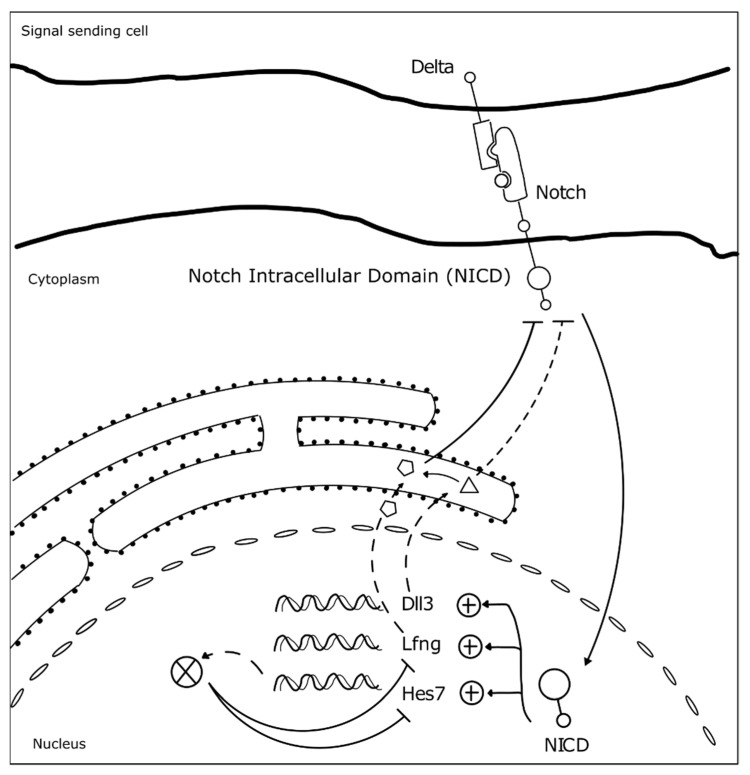
Negative feedback loops underlie somitogenesis clock oscillations. The identified mutated genes in SCDO are represented. Upon Notch receptor activation by a Delta ligand from a neighbor cell, the Notch intracellular domain (NICD) translocates to the nucleus where it activates the transcription of HES7, LFNG and DLL3 [23]. Lfng and Dll3 act cooperatively to repress further NICD activation [50], while Hes7 acts to represses its own expression and that of Lfng [23].

Somitogenesis clock gene expression oscillations are cell autonomous, maintained by negative feedback loops (Figure 3) (reviewed in [23]). The sum of the temporal delays involved in the process—from transcription, RNA splicing and decay, to protein translation and degradation—is crucial for robust oscillations to occur with a defined rhythm [31]. The deletion of all introns in mouse Hes7, for example, abolished oscillatory expression dynamics, causing sustained Hes7 expression in the PSM and severe vertebral defects [43]. Also, a reduction in the number of Hes7 introns led to accelerated oscillations and supernumerary somites, further evidencing the importance of the temporal delays imposed by mRNA splicing in periodic clock oscillations [44]. Another important feature is the mRNA and protein degradation rates. Nitanda and collaborators (2014) showed that the 3′UTR of clock mRNAs is a key defining feature for their expression patterns. By exchanging the 3′UTRs of Hes7 and Lfng, they found that the expression patterns obtained were dictated by the 3′UTR sequence per se, and not the gene it originally belonged to [45]. 3′UTRs are important to regulate the half-life of the respective mRNAs, partly because these regions are targeted by microRNAs, which can affect both the period and amplitude of the oscillations, or even lead to dampening and loss of the oscillatory dynamics [46,47]. The clock proteins’ half-life also plays an important role in the maintenance of the oscillatory dynamics [48]. Hirata and collaborators (2004) showed that increasing Hes7 half-life from ~20 to ~30 min lead to progressive dampening and loss of oscillations and, consequently, to somite segmentation defects [48]. Interestingly, it was recently shown that the differences in cell-autonomous oscillation periods of the segmentation clock between species can be explained by the difference in the kinetics of the biochemical reactions involved in the different time delays, and not by the gene sequences themselves [49]. 

At the tissue level, the molecular oscillator needs to be synchronized between neighboring cells, in order to allow the correct number of cells to simultaneously differentiate into a new somite. Work performed in mouse and zebrafish embryos showed that Delta-Notch signaling is required to couple oscillations between adjacent cells [51,52,53]. Additionally, oscillations of genes belonging to different signaling pathways need to be coupled. In fact, many clock genes are components of the Fgf, Wnt, and Notch signaling pathways [54,55], evidencing that the somitogenesis molecular clock is a complex network of oscillators, connected to ensure proper morphogenesis. For example, activation of Hes7 expression in the embryo tail bud is dependent on Fgf and not Notch signaling, which is then required to maintain Hes7 expression throughout the PSM. Hes7 in turn activates Fgf signaling modulators, such as the negative regulator Dusp4 [51]. Wnt signaling-dependent Axin2 also oscillates in the posterior PSM, out-of-phase relative to Lfng and Hes7 [39]. The coupling between Notch, Fgf, and Wnt signaling pathways in somitogenesis was beautifully evidenced by performing experiments with microfluidics devices, which showed that when Notch is entrained, Wnt expression dynamics is altered accordingly [56]. This is particularly important in the anterior PSM where the signaling pathways must converge to the same phase for proper somite segmentation [56].

Embryo clock synchronization between the left and right PSMs is crucial to ensure that somite segmentation occurs in a bilateral symmetric fashion. In zebrafish embryos Terra/Dmrt2a was shown to be necessary to maintain symmetrical clock gene expression patterns, as well as the same number of somites, in the left-right PSMs [57]. This role in left-right patterning is not conserved in the mouse embryo, but Dmrt2 was found to be indispensable for proper somite maturation [58] and formation of the vertebrae and ribs [59]. 

### 3.2. Somite Segmentation and Vertebrae Formation

**Figure 4:**
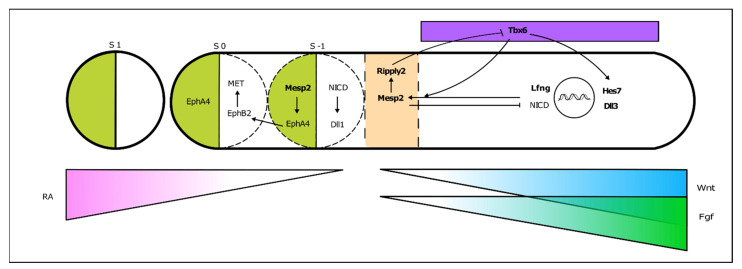
From somitogenesis clock oscillations to somite boundary formation. Periodic activation of Mesp2 at the rostral PSM, where FGF signaling is greatly decreased, triggers a molecular cascade ultimately leading to the budding off of a new somite. This is a simplified schematic representation, where the identified mutated genes in SCDO are highlighted.

PSM cells located posteriorly to the determination front are under the influence of high levels of Wnt and Fgf signaling and express the paraxial mesoderm marker Tbx6 (Figure 4). As the embryo elongates and cells in the anterior PSM leave the Fgf8 domain, Tbx6 and Notch are able to activate Mesp2 expression, delimiting in the caudal boundary of the next somite to be formed. Mesp2 plays a central role in the formation of the somitic boundary (reviewed in [60]). Mesp2 activates Ripply2 expression, which then represses Tbx6 [60,61]. Mesp2 also activates EphA4 which in turn induces the expression of EphrinB2 rostrally, in the caudal region of the somite-to-be. Here, EphrinB2 initiates a molecular cascade leading to rearrangements in the extracellular matrix [62] and to a mesenchymal-to-epithelial (MET) transition, culminating in somite boundary formation. EphA and EphrinB2 are then restricted to the rostral and caudal portion of the somite, respectively, ensuring proper somite polarity [63] (Figure 4).

**Figure 5:**
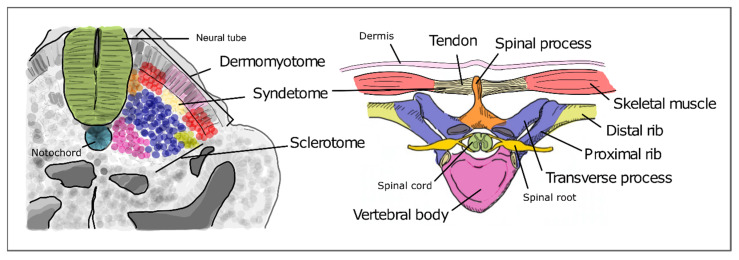
Differentiation of the different somite regions into definitive axial structures. After budding off from the PSM as epithelial spheres, somite differentiation takes place distinguishing the prospective cell populations that will give rise to adult structures. Colors in the figure represent precursor cells in the somite (**left**) and their respective definitive structures (**right**).

As somitogenesis progresses, the newly formed epithelial somites will undergo modifications and differentiate into multiple structures. Initially, the medial-ventral portion of the somite undergoes an epithelial-to-mesenchymal transition under the influence of Shh signaling emanating from the notochord and neural tube floor plate. This is now the sclerotome, containing the precursors of the vertebrae and ribs (Figure 5). The dorsal-lateral portion of the somite remains epithelial and expresses Pax3, under the influence of Wnt signaling from the dorsal neural tube—the dermomyotome. The latter then further differentiates into the myotome, which will originate the axial musculature and the dermatome, which will form the dermis of the back [32]. Pax3 activates Dmrt2/Terra expression in the dermomyotome [64], where it plays an important role in somite patterning and myogenesis [59,64]. 

The sclerotome undergoes a re-segmentation process essential for the final configuration of the vertebrae and all the structures of the spine. After re-segmentation, the caudal segment of a sclerotome joins the rostral segment of the adjacent sclerotome to form a vertebra. Due to this process, each segment of the adult axial skeleton is derived from a caudal and rostral part of two consecutive somites instead of one somite. The dermomyotome maintains the initial segmented configuration of the somites, allowing the derived muscles to intercalate each skeletal segment. This is crucial for the mobility and stability of the axial skeleton in humans. The most ventral portion of the sclerotome then surrounds the notochord to give rise to the body of the vertebrae and the intervertebral discs. The notochord gives rise to the nucleus pulposus of the intervertebral discs. The vertebral arch originates from the sclerotome cells that surround the neural tube. The ribs originate from the sclerotome cells located in the ventro-lateral region (Figure 5) [65,66]. 

## 4. Evidence for the Embryonic Origin of SCDO from Animal Models

As described in the previous sections, the genetic mutations identified in SCDO patients are in genes involved in the somitogenesis molecular clock—DLL3, LFNG, and HES7—and in the formation of the somite boundaries—MESP2, TBX6, and RIPPLY2. Each mutated gene is at the origin of different types of SCDO (described above). An additional mutation in DMRT2 has been reported associated with a phenotype that could represent a new type of SCDO [29]. Clues to how mutations in these genes lead to SCDO and other congenital malformations arise from Developmental Biology studies, where the role of these genes has been extensively studied in the context of vertebrate embryo body segmentation. Mutant mice experimental models have greatly contributed to understanding the role of specific genes in this process (as described in the previous section) and allow to study in further detail the molecular mechanisms that drive the onset of axial skeleton malformations. In fact, mice mutated in each one of the known genes that drive SCDO mostly recapitulate the phenotypes observed in Humans SCDO patients.

- The first Dll3 mouse mutants were generated in 1961, in a series of experiments using X-rays to induce gene mutations [67,68]. These mice, known as pudgy mice, presented shortened tails, trunks, and extensive axial skeleton malformations, similarly to what can be observed in patients with SCDO type 1. The skeleton defects were traced back to somitogenesis, where these mutants presented irregularly shaped and missing posterior somites, and somite formation was delayed [69]. The somitogenesis clock oscillations were perturbed in these mutants, as well the Mesp2 expression domains, which most probably underlies the phenotypes observed [69,70];
- Studies using Mesp2 mutant mice showed that it is essential for the formation of boundaries between adjacent somites in the anterior PSM. These mutants present defects in somite segmentation and rostral-caudal polarity, which ultimately lead to severe skeletal malformations across the axis extension, including rib fusions and abnormally shaped vertebrae and ribs [71]. The similarity between the skeletal defects in Human SCDO type 2 caused by MESP2 homozygous mutation and in the mouse embryo was further confirmed by three-dimensional computed tomography [72];
- In Lfng homozygous mutant mice, somitic boundaries are unclear, generating somites that are irregular both in size and shape. Hes7 is overexpressed along the PSM instead of presenting the typical dynamic patterns, although Notch signaling remained dynamic [73]. Dorsal-ventral somite patterning is also affected [74,75]. Consequently, the axial skeleton of these animals presents severe malformations, including incompletely formed vertebrae and vertebral and rib fusions. This homozygous mutation is usually deadly in the neonatal period due to respiratory problems driven by rib cage abnormalities;
- Heterozygous Hes7 mutant mice show kinked tails in 43% of the animals. The homozygous embryos presented severe defects of the axial skeleton, as found in type 4 SCDO. They had shorter trunks and tails and the majority died shortly after birth, apparently due to respiratory problems. Vertebrae and ribs were abnormally formed and vertebral bodies and neural arches were fused across the vertebral column [19]. Further analysis showed that Hes7 homozygous mutations lead to loss of Notch-dependent oscillatory expression of Lfng, NCID, and Nrarp. Interestingly, dynamic expression patterns of genes belonging to Fgf and Wnt signaling were maintained [73];
- Homozygous Tbx6 mouse mutants lack somites [76]. This is essentially because Tbx6 is required for paraxial mesoderm specification. In these mutants, there is no PSM and three neural tubes are formed instead. A careful analysis of the heterozygous mutants however, unveiled mild defects in the axial skeleton at E14,5 [76]. Additionally, heterozygous mutations of Tbx6 in rats can lead to skeletal malformations, including lumbar vertebral distortion and abnormal number of vertebrae, which resembles the autosomal dominant form of human SCDO type 5 [77];
- Ripply2 mutant embryos fail to form clear boundaries between somites, which also present polarity abnormalities and the homozygous mice die shortly after birth [78]. These mutants present severe axial skeleton malformations, including fused arches and pedicles. This phenotype is very similar to that found in Mesp2 mutants, possibly because both genes are involved in the same process of somite boundary formation;
- In 2006, Seo and collaborators engineered a Dmrt2 knock-out mouse in order to study the role of this gene during embryonic development. Homozygous mutants showed kinked tails and respiratory distress due to malformations of the thoracic cage [59]. Mutants had truncated ribs, rib bifurcations and fusions, along with other vertebral defects. Dmrt2 mutant mice die perinatally, similarly to the reported case of DMRT2-associated SCDO [59].

### 4.1. Environmental Contributions to SCDO

Besides the genetic background, the microenvironment experienced by the embryo may influence major steps of development, such as the formation of the axial skeleton [2]. Somite formation is highly regulated both in time and space and can be severely impacted by environmental factors. It has long been known that gestational hypoxia, for example, can lead to axial skeletal malformations [2]. Sparrow and collaborators (2012) further showed that gestational hypoxia combined with a pre-existing genetic risk for congenital scoliosis, significantly increased the penetrance and severity of the vertebral defects [79]. In fact, they found that the mild segmentation defects observed in heterozygous Hes7+/− and Mesp2+/− mutant mice, are severely exacerbated when the embryos experience short periods of hypoxia during gestation. The authors further showed that this was mediated by downregulation of Fgf8 expression in the PSM and consequent impairment of proper somite formation [79]. Consistent with this finding, in high-altitude geographic regions where atmospheric oxygen concentrations are lower, patients with congenital scoliosis tend to have a higher number and severity of rib deformities [80]. 

Besides external environmental factors, the health status and homeostasis of the mother can also affect embryo development. For example, the probability of fetus malformations increases up to 4-fold in diabetic mothers, and birth defects can be so severe that they lead to stillbirth [81]. Diabetes was recently validated as a risk factor for vertebral defects [82]. Indeed, a case of SCDO was reported in a newborn from a diabetic mother, along with other congenital malformations [83]. How elevated glucose levels in circulation impact embryo axial skeleton formation is still unclear. However, it was recently shown that the PSM presents a posterior-to-anterior gradient of glycolytic activity, which directly regulates Fgf and Wnt signaling gradients [84]. It is possible that the excess of glucose in the mother’s blood could affect this metabolic gradient, but this requires further investigation.

Further evidence that skeletal axis malformation could arise from a combination of environmental factors with genetic predisposition has been provided by studies in monozygotic twins. There are several reports of homozygous twins (identical genetic information) that displayed skeletal defects at different axial levels, or even where only one sibling presented vertebral defects [85,86,87]. This evidences the complex nature of the etiology of congenital axial skeleton defects, including SCDO, where both genetic and environmental factors are involved, many times in a synergistic manner.

### 4.2. New Insights on SCDO from Emerging Experimental Models

Although extraordinarily useful, animal models present important limitations when addressing human congenital disorders. Despite common genes and processes involved in the formation of equivalent morphological structures, there are species-specificities that cannot be circumvented. This is the case when studying embryo somitogenesis and axial skeleton formation, readily highlighted by the fact that the speed of somite formation (and overall biochemical reactions) is significantly slower in humans, when compared to mice or other animal model systems [49]. Additionally, although the same signaling pathways are implicated in the somitogenesis clock in multiple species, there is limited conservation of the specific genes that are involved [55]. The recent emergence of in vitro gastruloid and PSM-like organoid model systems derived from human embryonic stem cells or induced pluripotent stem cells have allowed the recapitulation of the initial stages of human development, such as gastrulation or even somitogenesis, with increasing similarity both at molecular and morphological levels [34,88,89]. Such systems were recently employed to study the impact of SCDO-associated mutations on human somitogenesis clock dynamics [34,88]. Two independent groups showed that a HES7 missense mutation, previously reported in type 4 SCDO patients, completely abolishes HES7-driven gene expression oscillations. Matsuda and collaborators further used induced pluripotent stem (iPS) cell lines derived from different patients with SCDO clinical features. In both cases, HES7 reporter activity showed sustained oscillations in 3D PSM-spheroids prepared from these cells. Mutations underlying the SCDO phenotypes were found in MESP2 and DLL3 genes. In the latter case, although 2D cultures showed clock oscillations, in the 3D culture system those were rapidly dampened, which is consistent with a requirement for Dll3 in maintaining the synchrony between cells [88]. These reports highlight the potential of these in vitro models to study the genetic mechanisms driving SCDO and the potential contribution of environmental factors to the onset of human vertebral defects. 

## 5. Conclusions

SCDO and other congenital axial skeleton disorders have compelled developmental biologists towards a deeper understanding of how the axial skeleton is formed during embryogenesis and, specifically, of the molecular and cellular events that are dysregulated in these situations. This is a paradigmatic example of how clinical and basic research, while pursuing fundamentally different approaches, can converge to better understand and respond to human congenital diseases. It is now clear that mutations in key genes participating in the somitogenesis clock and in somite boundary formation may lead to congenital malformations of the axial skeleton. However, much remains to be known about how each mutation elicits its characteristic phenotype and how different environmental factors may potentiate or even protect from more severe manifestations.

The current knowledge on the embryonic etiology of SCDO has greatly relied on the use of animal embryo models, and then cautiously inferred to be similar in Humans, at least to some extent. New emerging in vitro model systems are now allowing researchers to directly address the molecular and cellular processes occurring in the human system, without the technical and ethical constraints imposed on the use of human embryos. This opens the way for foreseeable significant advances in biomedical research in the near future.
